# Familial osteochondrodysplastic and cardiomyopathic syndrome in Chianina cattle

**DOI:** 10.1111/jvim.17221

**Published:** 2024-10-26

**Authors:** Joana G. P. Jacinto, Tolulope G. Ogundipe, Cinzia Benazzi, Irene M. Häfliger, Luisa V. Muscatello, Marilena Bolcato, Riccardo Rinnovati, Arcangelo Gentile, Cord Drögemüller

**Affiliations:** ^1^ Department of Veterinary Medical Sciences University of Bologna Bologna Italy; ^2^ Institute of Genetics, Department of Clinical Research and Veterinary Public Health, Vetsuisse Faculty University of Bern Bern Switzerland

**Keywords:** bovine, inbreeding, lethal allele frequency, precision medicine, recessive disorders

## Abstract

**Background:**

Skeletal dysplasia encompasses a heterogeneous group of genetic disorders characterized by an abnormal development of bones, joints, and cartilage. Two Chianina half‐sibling calves from consanguineous mating with congenital skeletal malformations and cardiac abnormalities were identified.

**Hypothesis/Objectives:**

To characterize the disease phenotype, to evaluate its genetic cause, and to determine the prevalence of the deleterious alleles in the Chianina population.

**Animals:**

Two affected calves, their parents and 332 Chianina bulls.

**Methods:**

The affected animals underwent clinicopathological investigation. Whole‐genome sequencing trio‐approach and PCR‐based assessment of the frequency of TDP‐glucose 4,6‐dehydratase (*TGDS*) and laminin subunit alpha 4 (*LAMA4*) alleles were performed.

**Results:**

The cases presented with retarded growth, poor nutritional status associated with muscular atrophy and angular deformities of the hindlimbs. Radiologic examination identified generalized osteopenia and shortening of the limb long bones. Necropsy showed osteochondrodysplastic limbs and dilatation of the heart right ventricle. On histological examination, the physeal cartilages were characterized by multifocal mild to moderate loss of the normal columnar arrangement of chondrocytes. Osteopenia also was observed. Genetic analysis identified a missense variant in *TGDS* and a splice‐site variant in *LAMA4*, both of which were homozygous in the 2 cases. Parents were heterozygous and allele frequency in the Chianina population for the *TGDS* variant was 5% and for the *LAMA4* variant was 2%.

**Conclusions and Clinical Importance:**

Genetic findings identified 2 potentially pathogenic alleles in *TGDS* and *LAMA4*, but no clear mode of inheritance could be determined.

**Abbreviations**
AI: artificial insemination
BTV: bluetongue virus
BVDV: bovine viral diarrhea virus
ECM: extracellular matrix
FAK: focal adhesion pathway
H&E: hematoxylin and eosin
IF: ichthyosis fetalis
IGV: Integrative Genomics Viewer
*LAMA4*
: laminin subunit alpha 4
PMT: pseudomyotonia congenita
RI: reference intervals
SBV: Schmallenberg virus
SDR: short chain dehydrogenase‐reductases family
*TGDS*
: TDP‐glucose 4,6‐dehydratase
VBG: venous blood gas analysis
WGS: whole‐genome sequencing

## 1 INTRODUCTION

Skeletal dysplasia comprises a heterogeneous group of rare genetic disorders characterized by abnormal development of bones, joints, and cartilage.^1^ Lethal dominantly inherited skeletal dysplasia syndromes in cattle include achondrogenesis type II *COL2A1‐*related in Holstein and crossbred (OMIA 001926‐9913),^2^, ^3^, ^4^, ^5^ chondrodysplasia *FGFR3*‐related (OMIA 001703‐9913),^6^ and vertebral and spinal dysplasia *T*‐related in Holstein (OMIA 001951‐9913),^7^ frontonasal dysplasia *ZIC2*‐related in Limousine (OMIA 002307‐9913),^8^ osteogenesis imperfecta type II *COL1A1*‐related in Red Angus and Holstein (OMIA 002127‐9913),^5^, ^9^, ^10^ Marfan syndrome *FBN1*‐related in Japanese Black (OMIA 000628‐9913),^11^ and skeletal‐cardio‐enteric dysplasia *MAP2K2*‐related in Romagnola (OMIA 002381‐9913).^12^ Lethal recessively inherited skeletal dysplasias also have been described in cattle. They include arachnomelia *MOCS1*‐ and *SUOX*‐related in Fleckvieh and Brown Swiss respectively (OMIA 001541‐9913; OMIA 000059‐9913),^13^, ^14^ brachyspina *FANCI*‐related (OMIA 000151‐9913)^15^ and complex vertebral malformation *SLC35A3*‐related (OMIA 001340‐9913)^16^ in Holstein, hereditary perinatal weak calf syndrome *IARS*‐related in Japanese Black (OMIA 001817‐9913),^17^ osteopetrosis *SLC4A2*‐related in Red Angus (OMIA 002443‐9913)^18^ and lethal multiorgan developmental dysplasia *KDM2B*‐related in Romagnola and Marchigiana (OMIA 001722‐9913).^19^, ^20^ Most of the skeletal dysplasias described in cattle are associated with malformations or defects in other organs such as the heart.

In recent decades, molecular investigations allowed the discovery of the etiology at the DNA level of an increasing number of diseases in cattle. This discovery also has occurred with respect to Chianina, 1 of the most important Italian autochthonous beef cattle breeds. Chianina cattle are mainly reared for beef production, and in recent years intensive selective breeding programs have been carried out to improve performance, leading to a worrisome increase in inbreeding rates.^21^ These practices have led to the emergence of 3 recessively inherited disorders: pseudomyotonia congenita (PMT; OMIA 001464‐9913),^22^ ichthyosis fetalis (IF; OMIA 002238‐9913),^23^ and ichthyosis congenita (OMIA 002450‐9913).^24^ Currently, PMT and IF genetic testing is routinely included in the breeding program of the Italian Association of Italian Beef Cattle Breeders.

Two half‐sibling Chianina calves with congenital skeletal malformations and cardiac abnormalities were identified. The aim of our study was to report the clinicopathological phenotype of a familial osteochondrodysplastic and cardiomyopathic syndrome in Chianina cattle. Moreover, genome analysis was carried out and the prevalence of possible deleterious alleles in the Chianina population was estimated.

## 2 MATERIALS AND METHODS

### 2.1 Animals

Our study did not require official or institutional ethical approval because it was not experimental, but rather part of clinical and pathological veterinary diagnostic testing. All animals in the study were examined with the consent of their owners and handled according to ethical standards. A total of 337 Chianina cattle, including 2 affected calves, their dams and their sire and 332 healthy control bulls were studied.

### 2.2 Clinical and pathological investigations

Two Chianina calves (cases 1 and 2) that were presented with obvious congenital skeletal malformations were referred to the Clinic for Ruminants of the University of Bologna. Both calves were the result of natural mating.

Case 1 was a 3‐month‐old female calf. It had a history of weakness, impaired growth and abnormal skeletal development. The calf underwent a physical examination and skeletal radiological investigation. In addition, venous blood gas analysis (VBG), CBC, serum biochemical analysis were performed. The calf was euthanized after the clinical investigations because of severe emaciation, lameness and renal disease, and submitted for necropsy and histological examination.

Case 2 was a stillbirth male calf reported because of skeletal abnormalities. After having carried out a postmortem skeletal radiological investigation, the animal was submitted for necropsy and histological examination.

The cases were tested for bovine viral diarrhea virus (BVDV), Schmallenberg virus (SBV), bluetongue virus (BTV), *Neospora caninum*, and *Toxoplasma gondii* using PCR and ELISA for detecting antigens and antibodies, respectively.

Tissues were collected, formalin‐fixed and paraffin embedded. Three‐micron thick sections were cut and routinely stained with hematoxylin and eosin (H&E). Bone tissue was further stained with toluidine blue.

### 2.3 Genetic analysis and DNA extractions

A pedigree analysis of the affected calves was performed.

Genomic DNA was obtained from the affected animals (ear cartilage tissue samples), their respective dams (EDTA blood samples) and the common sire (semen) using Promega Maxwell RSC DNA system (Promega, Dübendorf, Switzerland). Furthermore, genomic DNA also was obtained from semen of Chianina artificial insemination (AI) top sires' semen as well as from EDTA blood samples of Chianina young bull calves shortlisted for admission to the performance test at the testing station in the years 2017, 2018, 2019, and 2020.

### 2.4 Whole‐genome sequencing and variant calling

A whole‐genome sequencing (WGS) trio approach using the Illumina NovaSeq6000 (Illumina Inc., San Diego, CA, USA) was performed on the genomic DNA extracted from the 2 affected calves (cases 1 and 2), their 2 dams and the common sire. The sequenced reads were mapped to the ARS‐UCD1.2 reference genome, resulting in an average read depth of approximately 18.2× in case 1 and 17.9× in case 2, and single nucleotide variants and small indel variants were called.^25^ The applied software and steps to process fastq files into binary alignment map (BAM) and genomic variant call format files were in accordance with the 1000 Bull Genomes Project processing guidelines of run 7,^26^ except for the trimming, which was performed using fastp.^27^ Further preparation of the genomic data was performed as reported previously.^28^ The effects of the above variants were functionally evaluated with snpeff v4.3,^29^ using the National Center for Biotechnology Information Annotation Release 106 (https://www.ncbi.nlm.nih.gov/genome/annotation_euk/Bos_taurus/106/; accessed on 20 September 2022). This resulted in the final VCF file, comprising individual variants and their functional annotations. To identify private variants, we compared the genotypes of the cases with 942 cattle genomes of different breeds sequenced as part of the ongoing Swiss Comparative Bovine Resequencing project. Integrative Genomics Viewer (IGV)^30^ software version 2.0 was used for visual evaluation of genome regions containing potential candidate genes.

### 2.5 Homozygosity analysis

Biallelic variants were selected from the vcf file using PLINK v1.9^31^ command –biallelic‐only as a common quality control step. A genome‐wide search for homozygous regions shared by the 2 cases was performed using the R package detectRUNS v.0.9.6^32^ with the commands –*homozyg‐kb* 1000 (considering homozygous segments of at least 1000 kb), –*homozyg‐match* 0.95 (for allelic matching between both cases) and –*homozyg‐group* (for generating an overlap file), resulting in shared runs of homozygosity (ROH) indicating chromosomal region of identity‐by‐descent (IBD).

### 2.6 Variant validation and selection of candidate variants

The comprehensive variant catalog of run 9 of the 1000 Bull Genomes Project was used to investigate the allele distribution of variants within a global control cohort allowing the exclusion of common variants.^26^ The entire data set consists of 5116 cattle genomes including 576 from the Swiss Comparative Bovine Resequencing project, from a variety of breeds (>130 breeds indicated).

PredictSNP1,^33^ PolyPhen‐2,^34^ SIFT,^35^ and Mutpred2^36^ were used to predict the biological consequences of the detected missense variant. For cross‐species sequence alignments, the following NCBI protein accessions were considered: NP_001094629.1 (*Bos taurus*), NP_055120.1 (*Homo sapiens*), XP_522697.2 (*Pan troglodytes*), XP_001083495.1 (*Macaca mulatta*), XP_542640.3 (*Canis lupus*), NP_083854.3 (*Mus musculus*), XP_416988.1 (*Gallus gallus*), NP_956111.1 (*Danio rerio*), and XP_002939327.2 (*Xenopus tropicalis*).

The GeneMANIA tool^37^ was used to predict the interaction between TDP‐glucose 4,6‐dehydratase (*TGDS*) and laminin subunit alpha 4 (*LAMA4*).

### 2.7 Investigation of structural variants

To assess possible larger structural variants, including chromosomal, numerical, and structural abnormalities, the read depth along all chromosomes was calculated as previously described.^38^ In addition, in shared ROH identified regions (Table S3) the read depth also was calculated.

### 2.8 Sequence accessions

All references to the bovine *TGDS* gene correspond to the NCBI accessions NC_037339.1 (chromosome 12, ARS‐UCD1.2), NM_001101159.1 (*TGDS* mRNA), and NP_001094629.1 (TGDS protein). For the protein structure of TGDS the Uniprot database^39^ and InterPro^40^ with accession number A6QLW2 was used.

All references for the bovine *LAMA4* gene correspond to the NCBI accessions NC_037336.1 (chromosome 9, ARS‐UCD1.2), XM_024996678.1 (*LAMA4* mRNA), and XP_024852446.1 (LAMA4 protein). For the protein structure of LAMA4 the Uniprot database^39^ and InterPro^40^ with accession number A0A3Q1N9E9 were used.

### 2.9 Genotyping

Polymerase chain reaction and Sanger sequencing were used to confirm the WGS results and to perform targeted genotyping for the identified *TGDS* missense variant (12:69092831A>T). The 2 cases, their dams and the sire were genotyped. Additionally, Chianina AI top sires (n = 113) as well as Chianina young males shortlisted for admission to the performance testing at the testing station in the years 2017, 2018, 2019, and 2020 (n = 219) were genotyped. Primers were designed using the Primer‐BLAST tool. After amplification with AmpliTaqGold360Mastermix (Thermo Fisher Scientific), the purified PCR products were directly sequenced on an ABI3730 capillary sequencer (Thermo Fisher Scientific). The primer sequences used for genotyping the Chr12:69092831A>T varinat in *TGDS* were the following: 5′‐TCCAAGCCTATATCACACAGGT‐3′ (forward primer) and 5′‐GCCTACCACAGACAGTAAGT‐3′ (reverse primer). The primer sequences used for genotyping the Chr9:38176716GAGAAAGTGAGAGAGGGAAACAGAGGGGAGAGAGAA>G variant in *LAMA4* were the following: 5′‐AAAAGGAGAAAGCTGCCCAAG‐3′ (forward primer) and 5′‐CGGCCACTCTTAGTCCTTGC‐3′ (reverse primer). The sequence data were analyzed using Sequencher 5.1 software (GeneCodes).

## 3 RESULTS

### 3.1 Clinical phenotype

**FIGURE 1:**
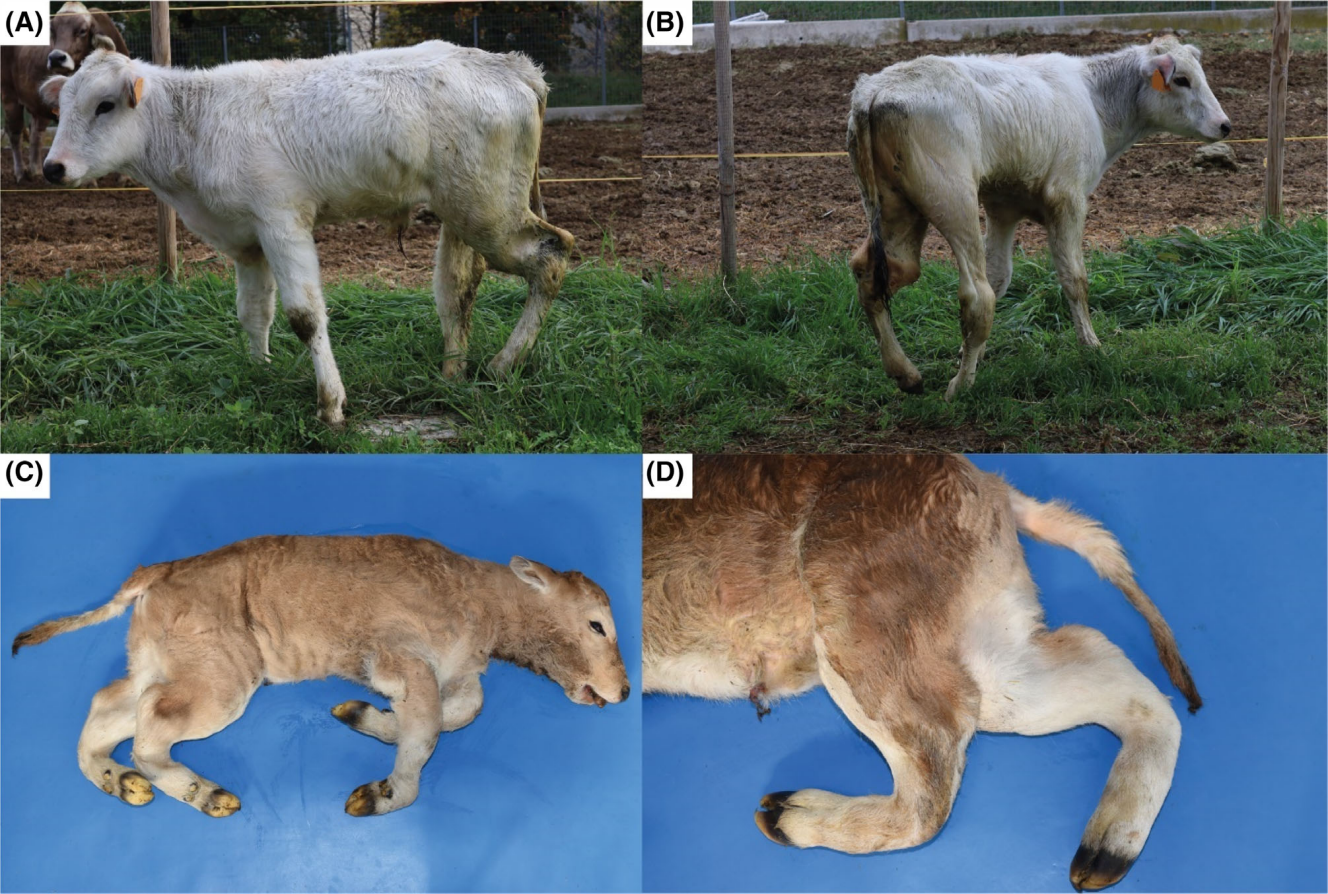
Osteochondrodysplasia in Chianina cattle. (A) and (B), Case 1 showing severe angular deformities of the hindlimbs mainly in the region of the hock joint and in the distal portion of the tibia. Note that the left hindlimb is more severely affected and consequently the calf only places it with the hoof tip. Note the pronounced varus posture of the hindlimbs. (C) Case 2 showing severe angular deformities of the hindlimbs in the region of the hock joint, in the distal portion of the tibia and proximal portion of the metatarsal bone. Note the inferior brachygnathia. (D) Case 2, detail of the hindlimbs.

On physical examination, case 1 had retarded growth and poor nutritional status associated with muscular atrophy and skeletal abnormalities (Figure 1A). These were characterized by pronounced varus of the hindlimbs and wide stance of the forelimbs. Severe angular deformities of the hindlimbs mainly in the region of the hock joint and in the distal portion of the tibia were noted (Video S1). In particular, the left hindlimb was more severely affected and consequently the calf only placed it with the hoof tip (Figure 1B). Limited motion of the hock join bilaterally but more severe in the left hindlimb was perceived on passive motion testing. The gait was impaired and characterized by reluctance to move and with complete weight transfer of the left to the contralateral hindlimb. The haircoat was dull with presence of *Linognathus* spp. The oculoconjunctival mucous membranes were pale and the episcleral vessels were visible. The heart rate was slightly increased at 90 beats/minute. Spontaneously voided urine was macroscopically pale yellow and transparent. The urine specific gravity was 1.010 and the pH 8.5.

Venous blood gas analysis yielded a temperature corrected pO_2_ of 37 mmHg and O_2_ saturation of 51.4%. A CBC showed moderate erythrocytosis (10.2 × 10^12^/L; reference intervals [RI]: 5‐7.2), with microcytosis (mean cell volume: 29.1 fL; RI: 38‐51), decreased mean corpuscular hemoglobin (10 pg; RI: 11‐17), but with normal hematocrit (31.9%; RI: 24‐35) and mean cell hemoglobin concentration (34.9 g/dL; RI: 34‐38). Echinocytes also were noticed in the blood smear. On plasma biochemistry, marked hyperlactatemia (7.2 mmol/L; RI: 0‐2), hypocholesterolemia (46 mg/dL; RI: 163‐397), increased BUN concentration (67 mg/dL; RI: 4.2‐20.4), increased uric acid concentration (1 mg/dL; RI: 0.1‐0.4) and mild hyperkalemia (6 mmol/L; RI: 3.9‐5.75) were identified.

In both cases, tests for BVDV, SBV, BTV, *Neospora caninum*, and *Toxoplasma gondii* were negative using PCR and ELISA.

### 3.2 Skeletal radiological investigation

**FIGURE 2:**
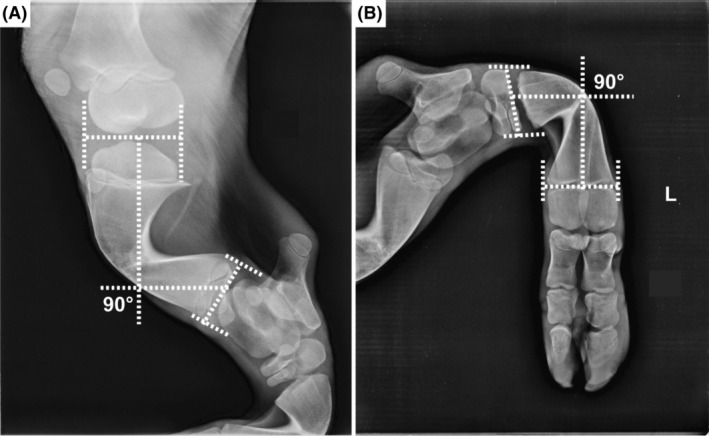
Radiology of the hindlimbs of Chianina cattle with osteochondrodysplasia. (A) Note the 90° angular deformity in the tibia. In the proximal tibia, secondary ossification centers appear to be narrowed and with slower development in Case 2. (B) Note the 90° angular deformity in the metatarsus in Case 2.

The radiological examination showed similar findings in both cases. The distal femur and proximal tibia secondary ossification centers appeared to be decreased and with slower development. The metaphysis of the same segments was enlarged and with sharp medial and lateral angles. The metaphyses maintained their internal areas of trabecular bone with vertical striations and, in the tibia, varus deformity was present (Figure 2A,B). Generalized osteopenia with cortical thinning also was present. The radius showed bone shortening with morphologic changes, and generalized osteopenia and cortical thinning were present. The metacarpal and metatarsal bones were shorter than normal, asymmetrically misshapen, with enlargement of proximal and distal ends and bending of the shafts. Cortical thinning was present.

### 3.3 Pathological phenotype

**FIGURE 3:**
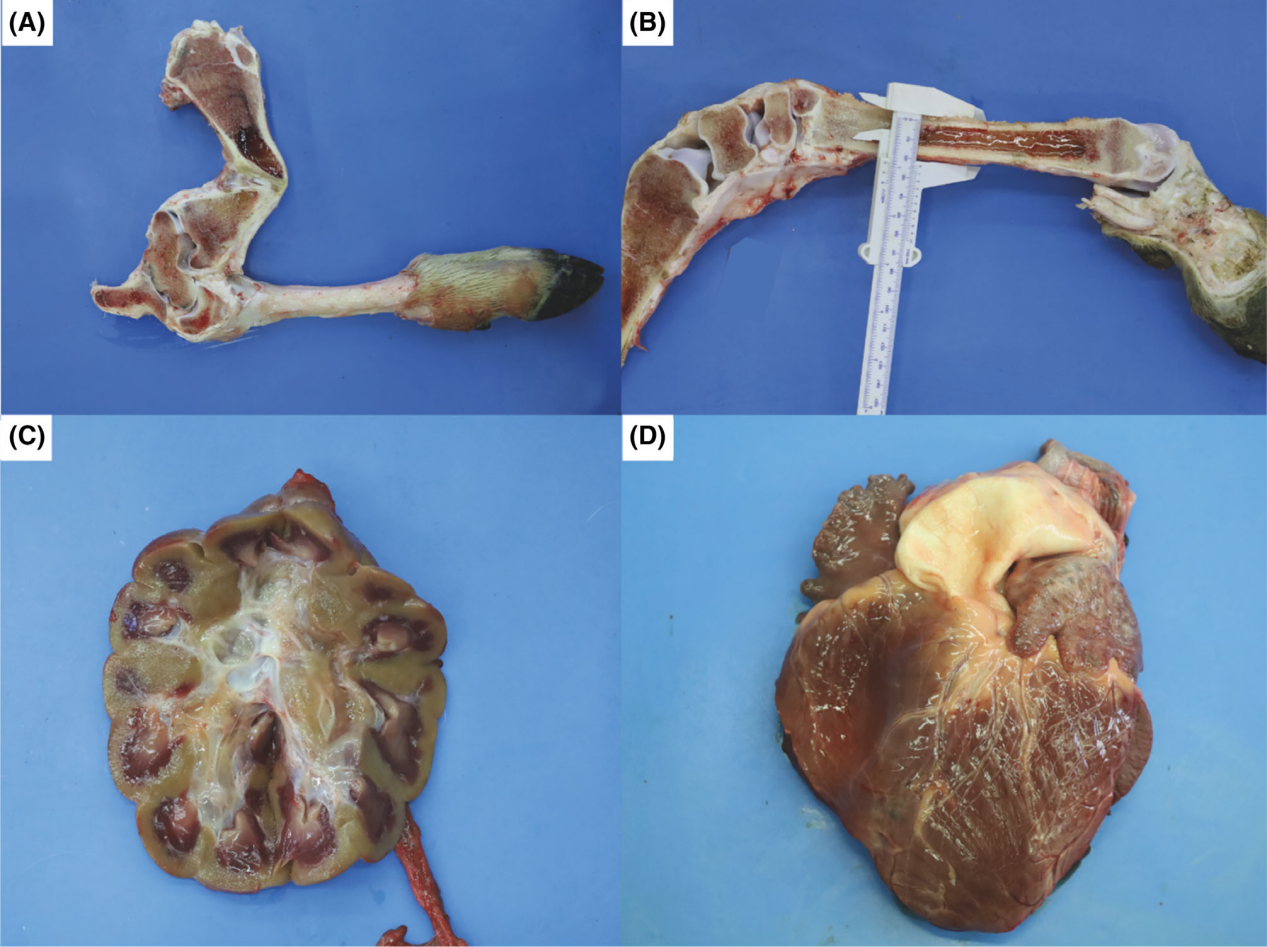
Gross pathological findings of Chianina cattle with osteochondrodysplastic and cardiomyopathic syndrome. (A) Sagittal section. The left tibia showing an angle of approximately 90° at the lower 3rd in the dorsoventral direction; the diaphyseal medulla is interrupted at the angle mentioned above in Case 1. (B) Sagittal section. The diaphyseal metatarsal is 2.5 cm thick, and the centrally located hematopoietic medulla has been replaced by adipose tissue in case 1. (C) Kidney showing a light hazel cortex in Case 2. (D) Heart showing a dilated and ectatic right ventricle and epicardium lining the left ventricle with ectatic lymphatics in Case 1.

Macroscopically, case 1 had localized hindlimb chondrodysplasia, with severe volume increase of the left tibio‐tarsal joint, and on the right a similar modest volume increase of the tibio‐tarsal joint accompanied by curvature of the tibia and metatarsus. In the left limb, there was evidence of edema infiltrating the gluteal muscles. The capsule of the left tibio‐tarsal joint had a thickness of 1 cm, and yielded approximately 10 mL of clear, colorless synovial fluid. The left tibia showed an angle of approximately 90° at the lower 3rd in the dorsoventral direction (Figure 3A). In sagittal section, the diaphyseal medulla was interrupted at the angle. The red pith typical of young subjects was present in the proximal segment for a length of 4 cm near the interruption, formed by a 0.5 cm layer of connective tissue. The diaphyseal metatarsal was 2.5 cm thick, and the centrally‐located hematopoietic medulla was replaced by adipose tissue (Figure 3B).

The kidneys had a light hazel cortex (Figure 3C). The heart had a dilated and ectatic right ventricle, whereas the epicardium lining the left ventricle showed ectatic lymphatics (Figure 3D).

Case 2 showed marked prognathism and micrognathia (Figure 1C). All 4 limbs were osteochondrodysplastic: the forelimbs curved and rotated inward, both at the height of the humerus; the hindlimbs also were intrarotated, T forming a 30° angle at the tarsometatarsal joint (Figure 1D). On section, the distal epiphysis of the femur showed a rounded portion of red hematopoietic marrow approximately 3 cm in diameter in a central position, as did the tibia. On sectioning, the tibia had hematopoietic marrow for approximately 2 cm in the central portion of the proximal epiphysis whereas the remainder appeared to consist of spongiosa. In the middle, the diaphysis had an angle of approximately 30° in the caudal position with an apposition of connective tissue of 1.5 cm. The macroscopic findings of the heart and kidneys were similar to those of case 1.

On histological examination of the heart, the connective tissue between the groups of cardiomyocytes was edematous with ectatic lymphatic vessels. Leakage of blood from small vessels with erythrocytes had accumulated in the spaces between the bundles of heart muscle fibers and infiltrated between individual fibers. Small foci of lymphoid cell myocarditis surrounded the capillaries and some of the arterioles, and were more prominent in thickness in the epicardium. The histomorphological features of the heart resembled a form of cardiomyopathy.

**FIGURE 4:**
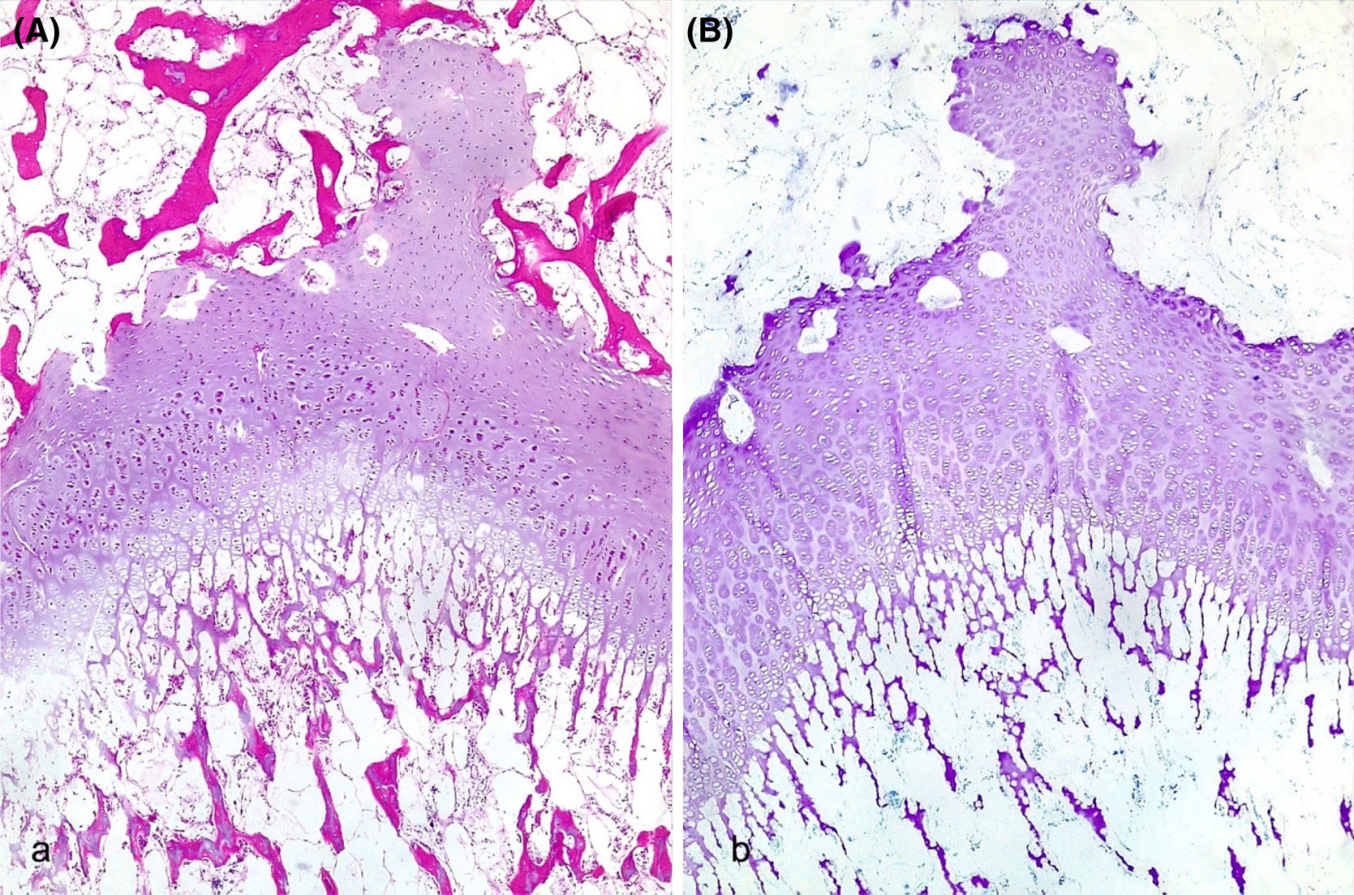
Histological findings of Chianina cattle with osteochondrodysplasia. (A) Physeal cartilage, 4×, hematoxylin and eosin: Loss of the columnar arrangement of chondrocytes, more severe in the proliferative zone in Case 2. (B) Physeal cartilage, toluidine blue, 4×: Uniform staining indicating no cartilage degeneration in Case 2.

In the long bones, the physeal cartilage, more severe in case 2, was characterized by multifocal mild to moderate loss of the columnar arrangement of chondrocytes. The chondrocytes of the proliferative zone were multifocal and arranged in clusters with loss of columnar polarity. The primary spongiosa was decreased in thickness with occasional retention of hypertrophic chondrocytes. In both cases, the metaphyseal trabeculae were severely thin and spaced apart, with retention of islands of cartilage in the center of the trabeculae, morphologically suggestive of bone rarefaction (osteopenia; Figure 4A). No cartilage matrix degeneration was observed with toluidine blue staining (Figure 4B). The morphological features of the bones were indicative of physeal osteochondrodysplasia.

### 3.4 Genetic analysis

**FIGURE 5:**
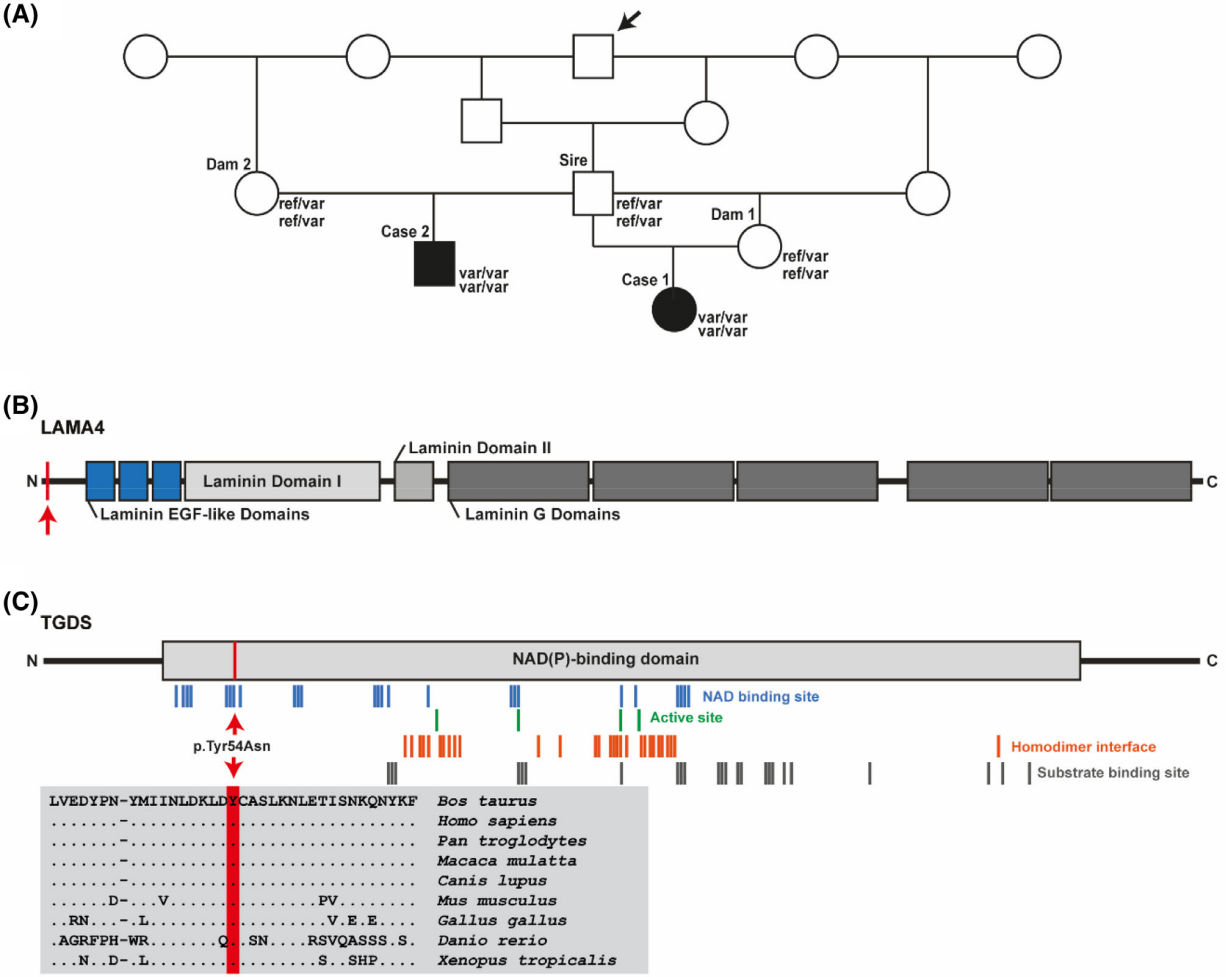
Homozygous laminin subunit alpha 4 (*LAMA4*) splice‐site and TDP‐glucose 4,6‐dehydratase (*TGDS*) missense variants in the 2 affected Chianina calves. (A) Pedigree of the 2 cases affected by the osteochondrodysplasitc and cardiomyopathic syndrome. Note the inbreeding loops between the parents. The arrow indicates the common ancestor. DNA samples available from animals with genotypes for both variants are given below the symbols. (B) Schematic representation of the bovine LAMA4 protein and its functional domains. Note that the *LAMA4* splice‐site variant is situated at the very beginning of the protein (red line and arrow). (C) Schematic representation of the bovine TGDS protein and its functional domains. Multiple sequence alignment of TGDS protein encompassing the region of the p.Tyr54Asn variant demonstrates complete evolutionary conservation across species.

Pedigree analysis indicated multiple inbreeding loops between the parents and a common male ancestor born in 2005 occurring 2‐3 generations previously (Figure 5A). Given the apparent consanguinity, the fact that both sexes are affected and that no parents showed clinically visible defects, we hypothesized that the current cases could be explained by a rare recessive inherited variant. In addition, because both cases were sired by the same bull, we also considered possible dominant inheritance. We therefore hypothesized 2 different possible scenarios: (i) a spontaneous, fully penetrant dominant trait arising from a de novo mutation inherited from the sire's germline or (ii) an autosomal recessive trait that was present in the homozygous state and inherited from both (heterozygous) parents.

Filtering of WGS for private shared heterozygous variants, present in the sequenced genomes of cases 1 and 2 and absent in 942 available control genomes (including the 3 parents), identified no variant common to both cases.

Filtering of WGS for private shared heterozygous variants present in the sequenced genomes of cases 1 and 2 and sire and absent in 942 available control genomes (including the 2 dams), identified 5 private protein‐changing variants predicted as moderate. Analysis of the occurrence of these variants in the global control cohort of 4540 genomes from a variety of breeds^26^ identified 3 remaining protein‐changing variants with predicted moderate impact that were heterozygous only in the genomes of cases 1 and 2 and their sire (Table 1). These 3 variants then were visually inspected using the IGV software (Broad institute, Cambridge, MA, USA), which confirmed 1 as a true variant. This heterozygous variant at Chr 7:72238027C>T was a missense variant in exon 19 of the *ATPase phospholipid transporting 10B* (*ATP10B*) gene (XM_015472347.2:c.3574G>A). It exchanges the encoded amino acid of ATP10B at position 1192 (XP_015327833.2: p.Asp1192Asn), located in the P‐type ATPase, C‐terminal domain. Its effect was predicted to be neutral (PredictSNP1 score 60% neutral; PolyPhen‐2 score 64% neutral; SIFT score 46% neutral).

**TABLE 1:** Results of variant filtering of the affected calves using the whole‐genome sequence data.

Filtering Step	Homozygous Variants	Heterozygous Variants
All variants in case 1	3 529 543	4 668 770
All variants in case 2	4 023 845	3 808 248
Private variants in cases 1 and 2	6743	0
Private variants in cases 1 and 2 and their sire	NA	558
Private variants in cases 1 and 2 with obligatory carrier parents (protein‐changing)	6180 (245)	NA
Private protein‐changing variants in cases 1 and 2 with obligatory carrier parents and absent in homozygous state in 942 controls	45	NA
Protein‐changing private variants in cases 1 and 2 with obligatory carrier parents and absent in homozygous state in a global cohort of 5279 controls	5	NA
Protein‐changing private variants absent in both parents (de novo mutations) and absent in 942 controls	NA	0
Protein‐changing private variants absent in the dams and present in the sire (de novo germinal in the sire) and absent in 942 controls	NA	5
Protein‐changing private variants absent in the dams and present in the sire (de novo germinal in the sire) and in a global cohort of 5279 controls	NA	3

Abbreviation: NA, not applicable.

Filtering of WGS for private shared homozygous variants, present in the sequenced genomes of cases 1 and 2, heterozygous in the 3 parents, and allowing the 942 available control genomes to be either heterozygous or homozygous wild type, identified 45 private protein‐changing variants with predicted moderate or high impact. Analysis of the occurrence of these variants in the global control cohort of 4540 genomes from a variety of breeds^26^ identified 13 remaining protein‐changing variants with predicted moderate or high impact that were homozygous only in the genomes of cases 1 and 2. These 13 variants then were visually inspected using the IGV software (Broad institute, Cambridge, MA, USA), which confirmed 12 as true variants (Table S1). Among these 12 remaining private variants involving 11 different genes, 2 variants affected interesting functional candidate genes for the studied phenotype. One was a homozygous variant at Chr12:69092831A>T that is a missense variant in exon 3 of the *TGDS* gene (NM_001101159.1: c.160T>A; Figure S1). It exchanges the encoded amino acid of TGDS at position 54 (NP_001094629.1: p.Tyr54Asn), located in the NAD(P)‐binding domain (Figure 5C). Furthermore, the tyrosine‐to‐ asparagine substitution affects a highly conserved residue (Figure 5D) and was predicted to be deleterious by 4 different tools (PredictSNP1 score 72% deleterious; PolyPhen‐2 score 68% deleterious; SIFT score 79% deleterious; Mutpred2 score 0.922 deleterious). Indeed, it is predicted to alter the ordered interface, the metal binding, the DNA binding and transmembrane protein, to cause loss of the allosteric site at p.Tyr54 and loss of staining, and to cause gain of relative solvent accessibility, of catalytic site at p.Asp50 and of acetylation at p.Lys59.^36^ The 2nd variant affecting an interesting functional candidate gene for the studied phenotype was a homozygous splice‐site variant (Chr9:38176716GAGAAAGTGAGAGAGGGAAACAGAGGGGAGAGAGAA>G) affecting exon 1 of the *LAMA4* gene (Figure 5B; Figure S2). Based on the used NCBI annotation it is predicted to be a splice‐site variant that most likely leads to the expression of an altered protein‐coding sequence (XM_024996678.1:c.153_153 + 34delAGTGAGAGAGGGAAACAGAGGGGAGAGAGAAAGAA).

Analysis of the remaining 9 identified variants, taking into account the known function of the genes, the reported association with Mendelian diseases, and the in silico assessment of the molecular consequences of the variants in the proteins, did not identify any other plausible cause for the observed phenotype (Table S2).

Homozygosity mapping identified 70 IBD segments >1 Mb and up to 3.1 Mb on 15 different chromosomes shared by the 2 sequenced cases and for which the 3 sequenced parents were heterozygous (Table S3). Two of these regions, a 1.8 Mb segment on chromosome 9 and a 2.1 Mb segment on chromosome 12, contained the genes *LAMA4* and *TGDS*, respectively. No evidence of structural variants was found when analyzing the read depth coverage obtained in the ROH regions, nor were any chromosomal abnormalities detected when analyzing the read depth coverage across the chromosomes.

Analysis of *TGDS* genotyping data confirmed that cases 1 and 2 were homozygous and the dams and sire were heterozygous for the detected *TGDS* variant (Figure S1). Furthermore, the genotyping of the Chianina top sires and young bulls shortlisted for admission to performance testing at the testing station identified 32 heterozygous carriers (Table 2; Figure S3). In addition, 1 top sire was homozygous for the variant allele in *TGDS* but homozygous for the reference allele at *LAMA4*.

**TABLE 2:** Association of the missense variant in *TGDS* and of the splice‐site variant in *LAMA4* with the osteochondrodysplastic and cardiomyopathic syndrome in Chianina cattle.

	Genotype *TGDS*			*Genotype LAMA4*		
	ref/ref	ref/var	var/var	ref/ref	ref/var	var/var
Affected calves	0	0	2	0	0	2
Obligate carriers^a^	0	3	0	0	3	0
Chianina bulls	296	32	1	321	11	0
Normal control cattle from various breeds	5279	0	0	5278	1^b^	0

Abbreviations: ref, reference allele; var, variant allele.

^a^ Parents of the affected calves;

^b^ From Shorthorn breed.

Analysis of *LAMA4* genotyping data confirmed that cases 1 and 2 were homozygous and the dams and sire were heterozygous for the detected *LAMA4* variant. Furthermore, genotyping of the same Chianina males identified 11 heterozygous carriers, but no animals homozygous for the variant *LAMA4* allele (Table 2; Figure S3).

The total allele frequency in the male Chianina population was 5% for the *TGDS* variant and 2% for the *LAMA4* variant.

Using the GeneMANIA tool,^37^ *TGDS* and *LAMA4* were predicted to interact through *SYMD3* and *TP53* (Figure S4). In particular, *TGDS* was predicted to interact and be coexpressed with *SMYD3*. In addition, *SMYD3* showed physical interactions with *TP53*. Finally, *TP53* had physical interactions and colocalized with *LAMA4*.

## 4 DISCUSSION

We completed a comprehensive clinical, histologic, and genetic evaluation of 2 Chianina calves with a familial osteochondrodysplastic and cardiomyopathic syndrome. We present evidence for the occurrence of a rare, possibly recessively inherited congenital syndrome that could be explained by the 2 identified homozygous loss‐of‐function variants in *TGDS* and *LAMA4*. The trio‐based WGS approach identified 12 protein‐changing variants that simultaneously were uniformly homozygous in the genome of the affected calves. After gene function analysis, taking into account the occurrence of the variant alleles in a global control cohort, the rarity and breed specificity of the variants, and in silico effect predictions, only the *TGDS* and *LAMA4* alleles were considered to be potential candidates to explain the observed congenital syndrome. We speculate that this congenital syndrome could have a recessive inheritance either caused by the *TGDS* or the *LAMA4* allele. Alternatively, a parallel homozygosity for the *TGDS* and *LAMA4* alleles could be considered a potential candidate genetic cause of the observed phenotype. The *TGDS* variant was identified only in Chianina cattle, whereas the *LAMA4* variant was identified in both Chianina and Shorthorn cattle. Alternatively, evaluation of possible paternal germinal mosaicism identified only a single heterozygous missense variant in *ATP10B* with no evidence of deleterious effects. This variant was present in the genomes of the 2 affected paternal half siblings as well as in the sperm‐derived DNA of their sire. Taking this into account, the heterozygous *ATP10B* variant seems less plausible as a cause of the observed phenotype.

*TGDS* encodes the dTDP‐D‐glucose 4,6‐dehydratase protein that is a member of the short chain dehydrogenase‐reductases family (SDR), which plays a pivotal role in nucleotide‐sugar metabolism.^41^ It is 1 of the 70 SDR genes the roles of which include the metabolism of retinoids, steroid hormones, and prostaglandins, as well as formation of the extracellular matrix (ECM).^42^ In humans, pathogenic variants in *TGDS* are associated with the recessive inherited Catel Mankze syndrome (OMIM 616145) that is characterized by bilateral clinodactyly, micrognathia digital syndrome, and in some cases cleft palate and heart malformations.^43^ Affected patients are either homozygous mutant or compound heterozygous for the *TGDS* variants.^43^, ^44^ We observed some similarities of Catel Mankze syndrome with the phenotype of our 2 cases, such as severe bone dysplasia and micrognathia.

*LAMA4* encodes the laminin subunit alpha 4 protein, which is a member of the most abundant noncollagenous protein laminins that are components of the ECM of the basement membrane.^45^, ^46^ LAMA4 is considered a signaling molecule and a multidomain glycoprotein.^47^ Additionally, it plays an important role in tissue development, cellular adhesion, and migration. LAMA4 adhesion to integrins triggers cell signaling, regulation of pathways, and regulation of tissue formation during embryogenesis such as in cartilage development.^48^, ^49^, ^50^, ^51^ Furthermore, 1 of the pathways in which *LAMA4* is involved is the focal adhesion pathway (FAK). Interestingly, FAK signaling influences Wnt/β‐catenin pathways, impacting chondrogenesis and bone development.^52^, ^53^, ^54^ Disruption of this pathway may result in osteoarthritis, intervertebral disc degeneration, and rheumatoid arthritis.^54^, ^55^, ^56^ In humans, a missense variant in *LAMA4* has been associated with a dominant inherited form of dilated cardiomyopathy (OMIM 600133).^49^ We observed cardiac abnormalities sharing some similarities with dilated cardiomyopathy in humans such as dilatation of the heart and ectatic right ventricles in our cases. In addition, disruption in the FAK pathway could be caused by the observed splice‐site variant in *LAMA4* and impact bone development, contributing to the observed bone malformations.

We have described an inherited familial syndrome in cattle that may be associated with 2 independent deleterious recessive alleles in 2 different genes on different chromosomes. The central hypothesis of the Human Undiagnosed Diseases Network is that many of these rare genetic disorders remain undiagnosed because they are caused by multiple variants in >1 gene.^57^ Recent studies have identified a role for digenic inheritance of Mendelian diseases in humans^58^ and similar examples may exist in domestic animals.^59^ In humans, digenic autosomal recessively inherited diseases include the severe dilated cardiomyopathy, which occurs because of variants in *MYH7* and *TNNT2*.^60^ People who are homozygous for only 1 of the 2 pathogenic alleles do not have the disease. However, the disease is manifested when both variant alleles are present in parallel.^60^ Additionally, Bardet Biedl syndrome (OMIM 617119), a recessively inherited multiorgan syndrome, also can be digenically inherited. Clinical features of this syndrome include polydactyly, neurodevelopmental disabilities, obesity, rod‐cone dystrophy, speech deficit, and renal disease.^61^, ^62^, ^63^ In our study, only the 2 affected calves were homozygous for both the *TGDS* and *LAMA4* variants. Although 1 top sire was identified as a homozygous carrier for the *TGDS* variant, the bull was homozygous wild type for the *LAMA4* variant. We therefore hypothesized that, as in the above‐mentioned diseases of humans, possibly homozygosity for both *TGDS* and *LAMA4* variants would be required to manifest the congenital syndrome in cattle. Therefore, a combined or additive effect of both variants leading to an osteochondrodysplastic and cardiomyopathic syndrome might be plausible. Digenic forms of inheritance involving *TGDS* and *LAMA4* have never been reported. However, with the evidence presented in our study, it cannot be excluded that the presence of the homozygous variant in *LAMA4* or alternatively in *TGDS* itself might be causative. Therefore, mating of carriers should be avoided to prevent further similarly affected offspring. Future research will be needed to provide additional insight into the biological effects of the identified *TGDS* and *LAMA4* variants.

Currently, the use of short‐read WGS still has some limitations, including methodological limitations such as structural variant detection, in addition to the still incomplete annotation of the bovine genome. In fact, the annotation of bovine *LAMA4* is not entirely clear, as the alternative annotation available in the Ensembl genome browser shows that the deletion is in the 5′ untranslated region of the gene. Nonetheless, as RNA sequencing data shows reads in the region of the deletion, we believe that the variant allele might be functionally important, with a regulatory effect, or as a true coding variant as mentioned above. Other possible limitations are the focus on coding variants in selected candidate genes based on literature searches, challenges associated with the short‐read WGS approach, inaccuracies in read alignment and variant calling,^64^ and potential influences of epigenetics factors.

Moreover, collecting additional similarly affected calves could help confirm or rule out the possible causality of the 2 identified variants of *TGDS* and *LAMA4*.

Our study alerts veterinarians and breeders of Chianina cattle to the potential emergence of rare diseases in the future. Reporting of additional similarly affected calves would help in understanding the underlying possible genetic causes and better clarify the mode of inheritance and role of the identified variants.

## CONFLICT OF INTEREST DECLARATION

Authors declare no conflicts of interests.

## OFF‐LABEL ANTIMICROBIAL DECLARATION

Authors declare no off‐label use of antimicrobials.

## INSTITUTIONAL ANIMAL CARE AND USE COMMITTEE (IACUC) OR OTHER APPROVAL DECLARATION

This case description was not based on an invasive animal experiment but on clinical examination and diagnostic evaluation of spontaneous occurring cases; therefore, there are no associated authorization numbers. The control cattle sampling was performed for diagnostic purposes at admission at the testing station.

## HUMAN ETHICS APPROVAL DECLARATION

Authors declare human ethics approval was not needed for this study.

## Supporting information


**Table S1.** List of the remaining homozygous variants after comparison with the global control cohort of 5279 control genomes from other breeds and after IGV visual inspection, identifying 10 homozygous protein‐changing variants with a predicted moderate and high effect present only in the affected calves.

**Table S2.** Pathogenicity prediction results for the 12 homozygous protein‐changing variants exclusively present in the genome of the affected calves and absent in the global control cohort of 5279 genomes of a variety of breeds.

**Table S3.** Homozygosity mapping in the 2 cases.

**Figure S1.** Homozygous *TGDS* missense variant in the 2 affected Chianina calves. (A) *TGDS* gene structure showing the variant location on chromosome 12, exon 3 (red arrow). (B) IGV screenshot presenting the Chr12: g. 69092831A>T variant homozygous in the affected calves (shown below) and heterozygous in their parents identified by whole‐genome sequencing. (C) Electropherograms showing the normal, carrier, and case genotypes obtained by Sanger sequencing.

**Figure S2.** Homozygous *LAMA4* splice‐site variant in the 2 affected Chianina calves. (A) *LAMA4* gene structure showing the variant location on chromosome 9, exon 1 (red arrow). (B) IGV screenshot presenting the Chr9: g.38176716GAGAAAGTGAGAGAGGGAAACAGAGGGGAGAGAGAA>G variant homozygous in the affected calves (shown below) and heterozygous in their parents identified by whole‐genome sequencing.

**Figure S3.** Prevalence of carriers for the *TGDS* missense variant and *LAMA4* splice‐site variant causing osteochondrodysplastic and cardiomyopathic syndrome in Chianina. Note that the presented information is based on the number of young bulls eligible for admission to the performance test and sorted by year of birth and in the listed artificial insemination top sires.

**Figure S4.** Prediction of the interaction between *TGDS* and *LAMA4* using the GeneMANIA tool.

**Video S1.** Case 1 showing severe angular deformities of the hindlimbs mainly in the region of the hock joint and in the distal portion of the tibia. Note that the left hindlimb is more severely affected and consequently the calf places it only with the hoof tip. Note the marked varus posture of the hindlimbs.
