# Supplementary material for: Familial osteochondrodysplastic and cardiomyopathic syndrome in Chianina cattle
Source: J Vet Intern Med. 2024 Oct 26;38(6):3346–57. doi: 10.1111/jvim.17221 (PMC11586572; doi:10.1111/jvim.17221)
Supplement: Supplementary file 2 — Table S2. Pathogenicity prediction results for the 12 homozygous protein‐changing variants exclusively present in the genome of the affected calves and absent in the global control cohort of 5279 genomes of a variety of breeds. [file JVIM-38-3346-s004.docx]

**Supplementary Table S2.** Pathogenicity prediction results for the 12 homozygous protein-changing variants exclusively present in the genome of the affected calves and absent in the global control cohort of 5279 genomes of a variety of breeds.

| **Gene** | **OMIM** | **Associated disorder/ gene function** | **Protein change** | **Var/Ref** | **Breed occurrence** | **Predicted effect^1^** |
| --- | --- | --- | --- | --- | --- | --- |
| LOC100336282 | NA | Uncharacterized | p.Ala220Val | 4 | Gelbvieh, Marchigiana, Pajuna, Romagnola | NA |
| *KLHL33* | NA | Role in extracellular functions, morphology, and binding to other proteins | p.Tyr754Cys | 1 | Romagnola | neutral |
| *PARP2* | 607725 | Modify nuclear proteins by poly-ADP-ribosylation | p.Thr197Arg | 4 | Romagnola, Hanwoo, Corriente | neutral |
| *OR11G2* | NA | Olfactory receptor | p.Thr138Ala | 9 | Romagnola, Busa, Menggu, Mongolian, Texas Longhorn, Corriente | neutral |
| LOC783885 | NA | Olfactory receptor | p.Phe172Va | 4 | Romagnola, Busa, Corriente | neutral |
| *LCN6* | 609379 | May play a role in male fertility | p.Arg90Cys | 5 | Chianina, Hanwoo | neutral |
| *CCDC183* | 615955 | NA | p.Ala112Thr | 6 | Chianina, Norwegian Red, Luxi | neutral |
| *MAMDC4* | 617208 | Involved in the sorting and selective transport of receptors and ligands across polarized epithelia | p.Pro479Leu | 6 | Chianina, Hanwoo, Luxi | neutral |
| *DCT* | 191275 | Oculocutaneous albinism, type VIII | p.Phe429Cys | 2 | Marchigiana | neutral |
| *TGDS* | 616146 | Catel-Manzke syndrome, | p.Ser127Asn | 3 | Kalmykian, Hanwoo, Mongolian | neutral |
| *TGDS* | 616146 | Catel-Manzke syndrome | p.Tyr54Asn | 0 |  | deleterious |
| *LAMA4* | 600133 | Cardiomyopathy, dilated, 1JJ |  | 1 | Shorthorn | NA |
