# Supplementary figures and images for: Familial osteochondrodysplastic and cardiomyopathic syndrome in Chianina cattle
Source: J Vet Intern Med. 2024 Oct 26;38(6):3346–57. doi: 10.1111/jvim.17221 (PMC11586572; doi:10.1111/jvim.17221)

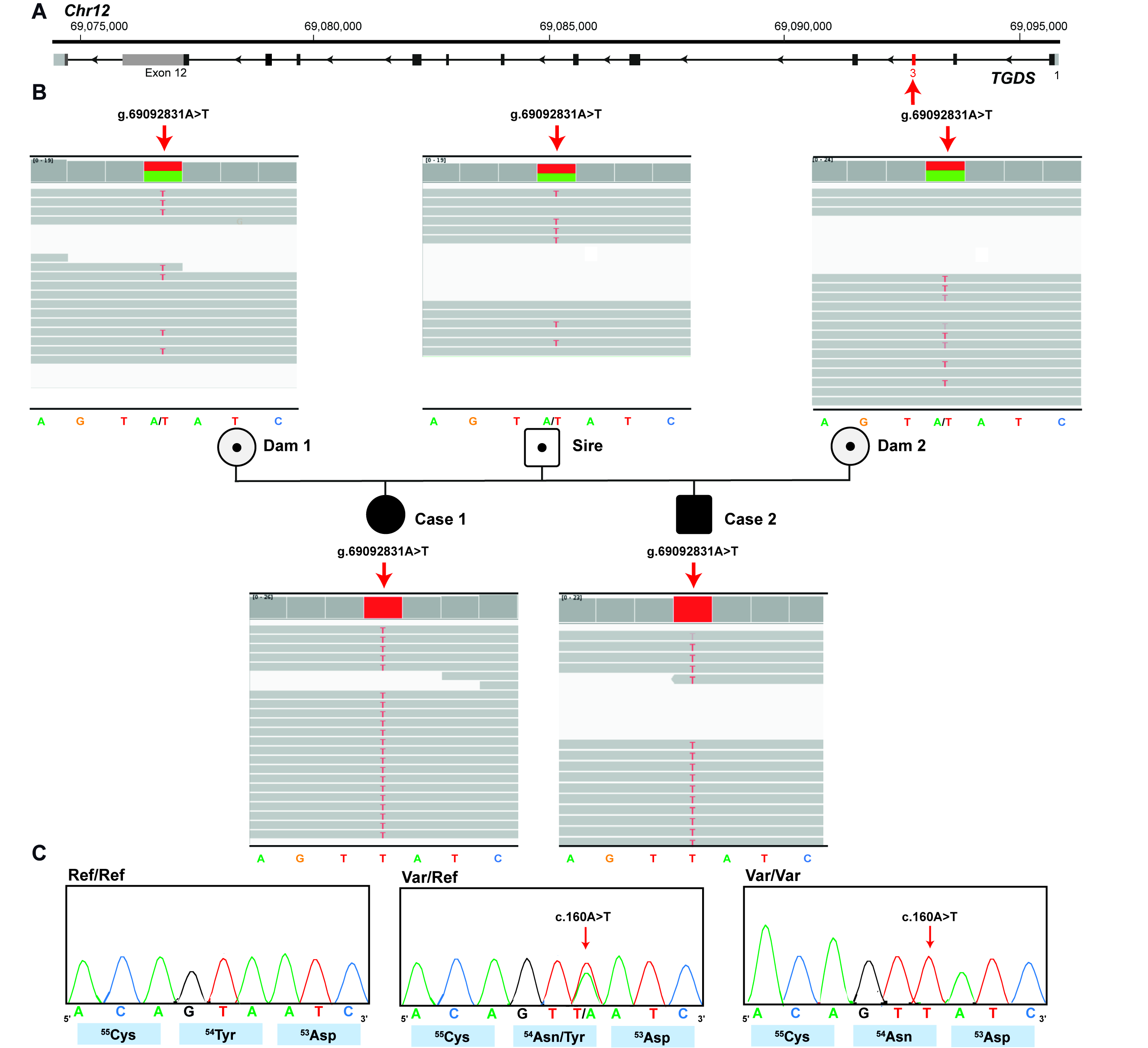

Supplement: Supplementary file 4 — Figure S1. Homozygous TGDS missense variant in the 2 affected Chianina calves. (A) TGDS gene structure showing the variant location on chromosome 12, exon 3 (red arrow). (B) IGV screenshot presenting the Chr12: g. 69092831A>T variant homozygous in the affected calves (shown below) and heterozygous in their parents identified by whole‐genome sequencing. (C) Electropherograms showing the normal, carrier, and case genotypes obtained by Sanger sequencing. [file JVIM-38-3346-s005.tif]

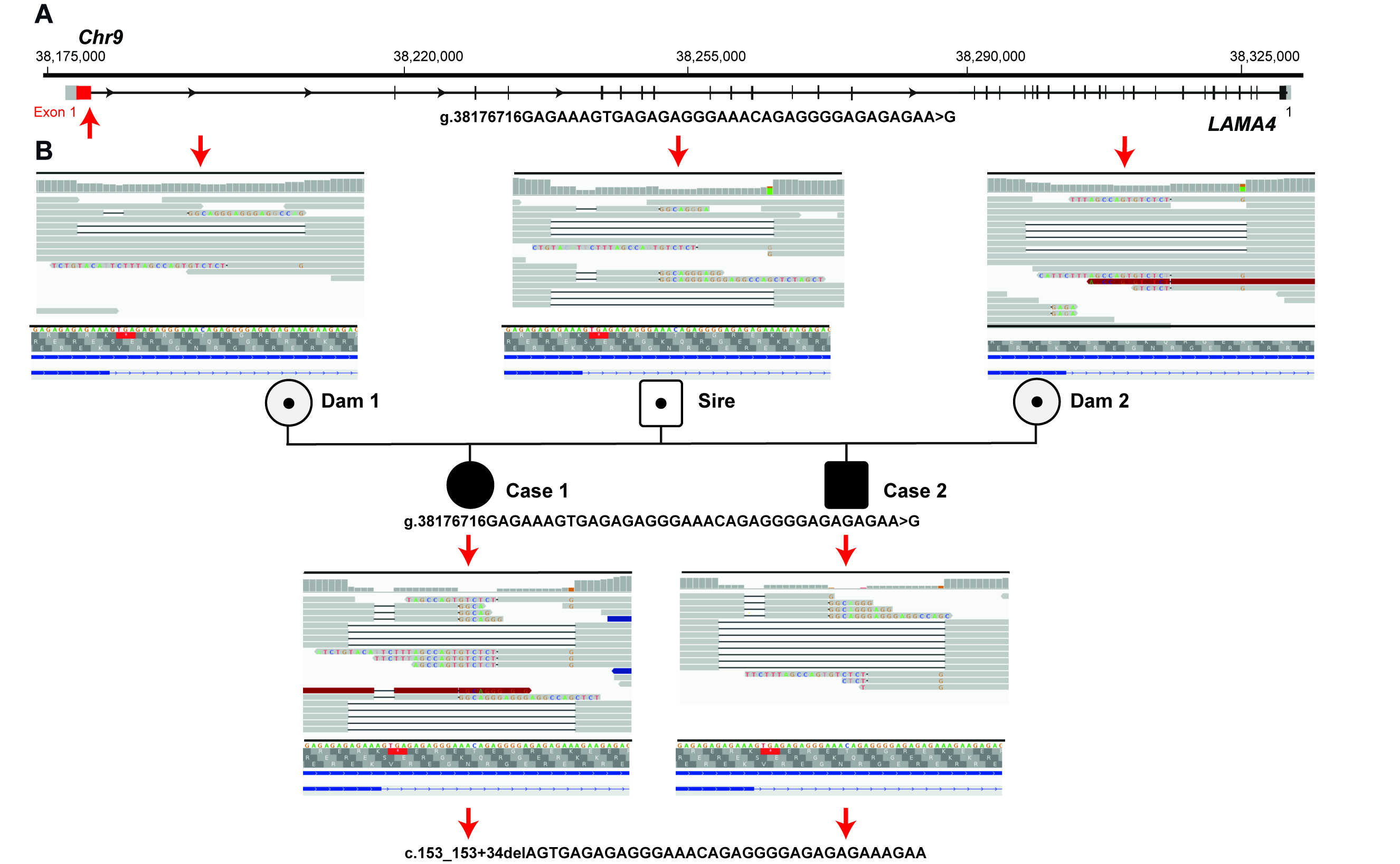

Supplement: Supplementary file 5 — Figure S2. Homozygous LAMA4 splice‐site variant in the 2 affected Chianina calves. (A) LAMA4 gene structure showing the variant location on chromosome 9, exon 1 (red arrow). (B) IGV screenshot presenting the Chr9: g.38176716GAGAAAGTGAGAGAGGGAAACAGAGGGGAGAGAGAA>G variant homozygous in the affected calves (shown below) and heterozygous in their parents identified by whole‐genome sequencing. [file JVIM-38-3346-s007.tif]

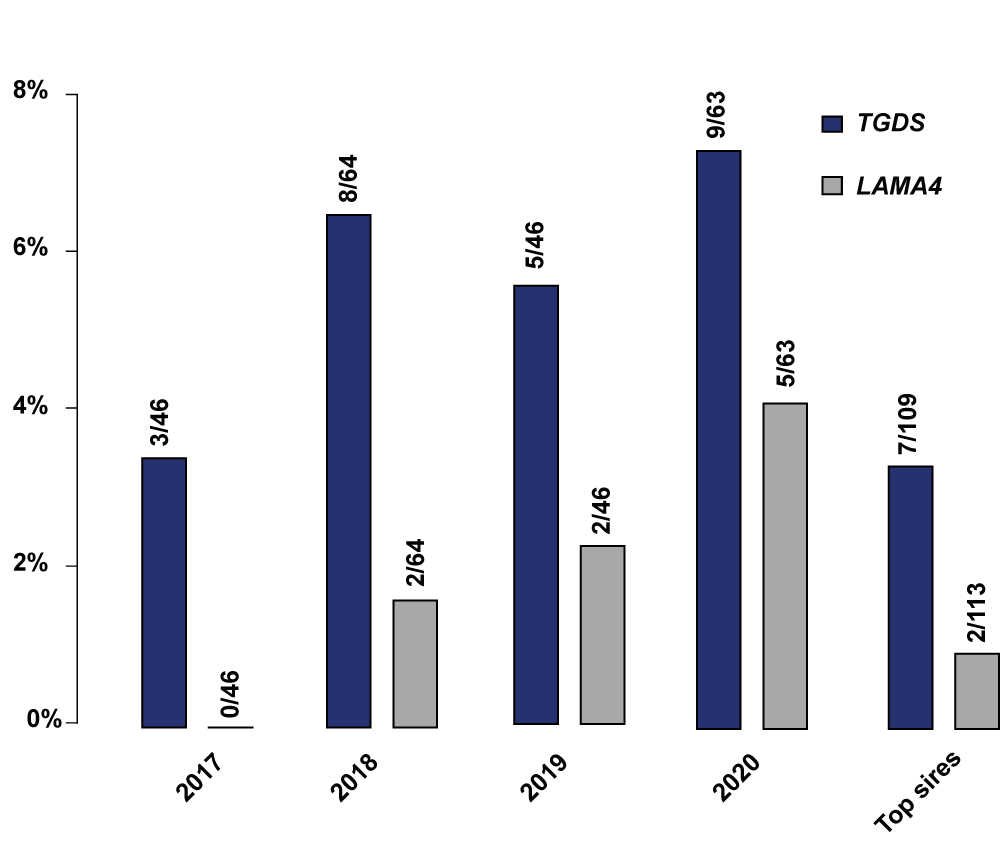

Supplement: Supplementary file 6 — Figure S3. Prevalence of carriers for the TGDS missense variant and LAMA4 splice‐site variant causing osteochondrodysplastic and cardiomyopathic syndrome in Chianina. Note that the presented information is based on the number of young bulls eligible for admission to the performance test and sorted by year of birth and in the listed artificial insemination top sires. [file JVIM-38-3346-s002.tif]

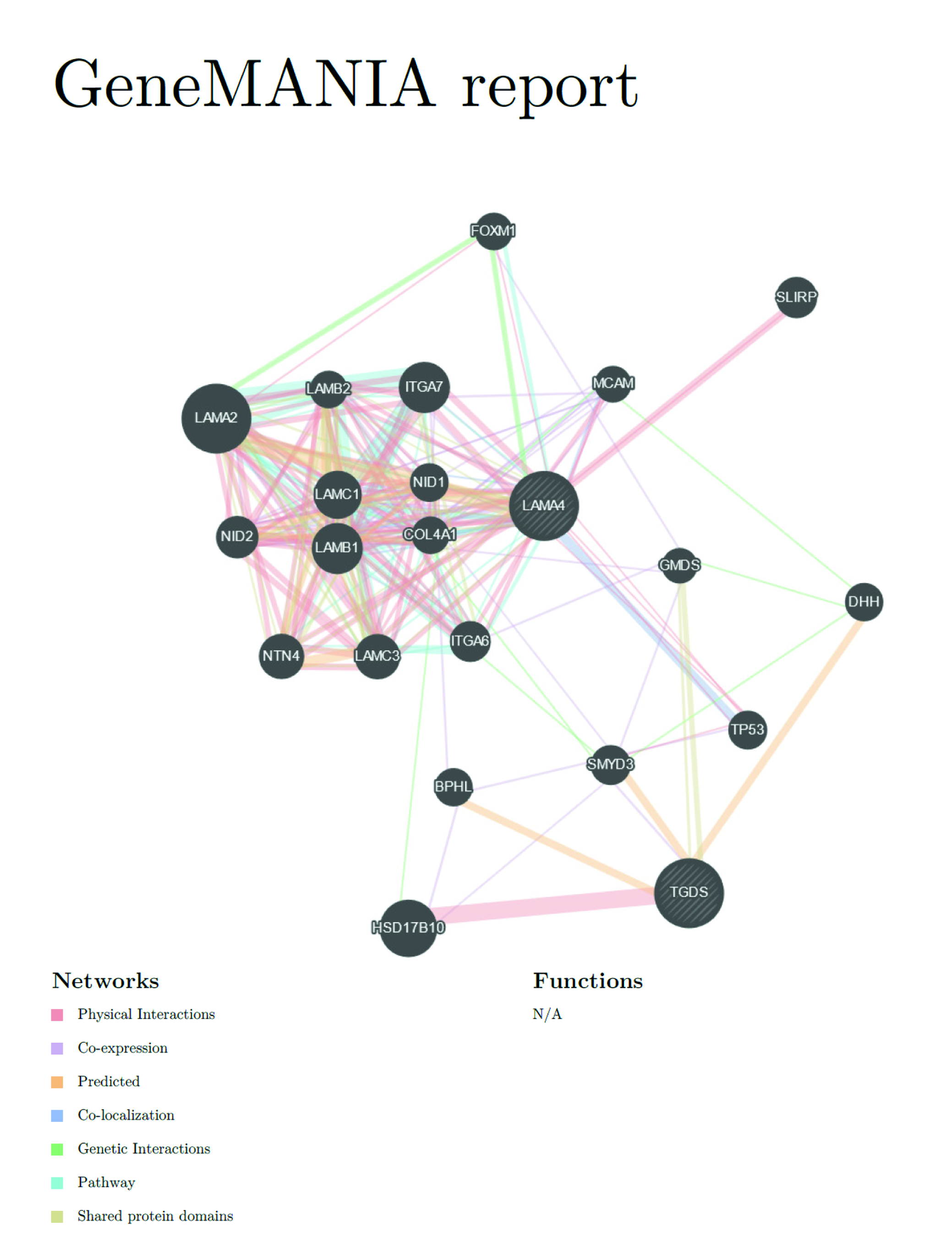

Supplement: Supplementary file 7 — Figure S4. Prediction of the interaction between TGDS and LAMA4 using the GeneMANIA tool. [file JVIM-38-3346-s001.tif]
